# Periprocedural considerations of transcatheter aortic valve implantation for anesthesiologists

**DOI:** 10.15171/jcvtr.2016.10

**Published:** 2016-06-28

**Authors:** Ata Hassani Afshar, Leili Pourafkari, Nader D Nader

**Affiliations:** ^1^Department of Anesthesiology, University at Buffalo, Buffalo, NY, USA; ^2^Cardiovascular Research Center, Tabriz University of Medical Sciences, Tabriz, Iran

**Keywords:** Aortic Valve Stenosis, TAVR, General Anesthesia

## Abstract

Transcatheter aortic valve replacement (TAVR) is rapidly gaining popularity as a viable option in the management of patients with symptomatic aortic stenosis (AS) and high risk for open surgical intervention. TAVR soon expanding its indications from "high-risk" group of patients to those with "intermediate-risk". As an anesthesiologist; understanding the procedure and the challenges inherent to it is of utmost importance, in order to implement optimal care for this generally frail population undergoing a rather novel procedure. Cardiac anesthesiologists generally play a pivotal role in the perioperative care of the patients, and therefore they should be fully familiar with the circumstances occurring surrounding the procedure. Along with increasing experience and technical developments for TAVR, the procedure time becomes shorter. Due to this improvement in the procedure time, more and more anesthesiologists feel comfortable in using monitored anesthesia care with moderate sedation for patients undergoing TAVR. A number of complications could arise during the procedure needing rapid diagnoses and occasionally conversion to general anesthesia. This review focuses on the periprocedural anesthetic considerations for TAVR.

## Introduction

The number of elderly patients presenting with clinically significant aortic stenosis (AS) has been rising in the developed countries.^1^ In fact, AS is the most prevalent form of valvular heart diseases in the western world and among elderly population.^2^ As medical treatment has little to offer for symptomatic AS, surgical intervention remained the only available option for years. In several frail elderly patients, the risk of perioperative mortality or morbidity becomes prohibitive of surgical replacement of the stenosed valve. While advanced age per se is not a solid contraindication for the surgical replacement of the aortic valve; age-associated comorbidities often necessitate shorter duration of operation with minimal valvular manipulation.^3^ With the introduction of transcatheter aortic valve replacement (TAVR), a new exciting era in the management of patients with symptomatic AS and high risk for open surgical intervention started.^4^ TAVR is a minimally invasive procedure that has been shown to have superior patient outcome compared to medical management of the patients with aortic stenosis, and non-inferior patient outcome compared to conventional surgical replacement of the aortic valve.^5^ Furthermore, there is a recent trend in offering TAVR for intermediate risk patients as this procedure was reserved only for inoperable, “high-risk” patients. The use of TAVR for intermediate-risk patients with AS is gaining more popularity as the reported rates of stroke and postprocedural mortality are lower than those undergoing surgical aortic valve replacement (AVR). TAVR might become the preferred treatment modality even for patients at intermediate-risk for open surgery, in the near future.^5^

Considering the medical, surgical and procedural challenges during the procedure, a multidisciplinary approach is recommended for a successful outcome. Cardiac anesthesiologists generally play a pivotal role in the perioperative care of the patients, and therefore they should be fully familiar with the circumstances occurring surrounding the procedure. This review focuses on the periprocedural anesthetic considerations for TAVR.

## Transcatheter valve types

Food and Drug Administration (FDA) has currently approved two trans-catheter valves to be used in severe aortic stenosis; the balloon-expandable SAPIEN valve (Edwards Lifesciences) and the self-inflating CoreValve (Medtronic, Inc.).^6^ SAPIEN valve is constructed of trileaflet bovine pericardium on a metal stent and its deployment requires rapid ventricular pacing during balloon inflation.^7^ The CoreValve is a trileaflet porcine pericardial valve mounted on a Nickel-Titanium based alloy (Nitinol) self-expanding stent and its insertion usually does not require rapid ventricular pacing.^8^ Both of these hardware choices have undergone vigorous evaluation in landmark clinical trials prior to obtaining commercial approval.^9,10^ Based on the results of CHOICE trial, which has examined both clinical and echocardiographic outcomes, there is no difference in the clinical outcomes between the patients implanted transfemorally with either Medtronic CoreValve or SAPIEN valve one year after TAVR.^11^ New generations of the current valves along with other technologies are evolving and awaiting approvals through clinical trials. There are different approaches for the deployment of these valves. The most widely practiced deployment methods for SAPIEN valves include transfemoral retrograde and transapical antegrade approaches.^7^ While CoreValve has been deployed via transfemoral, subclavian, axillary or direct aortic approaches.^12^ Aside from known complications, each approach for deployment is associated with a particular set of complications. To name a few, vascular injuries are associated in transvascular approaches (i.e., transfemoral and trans-axillary/subclavian approaches). There is a potential risk of lung injury and cardiac tamponade during transapical TAVR through a mini-thoracotomy approach.^12^

## Preoperative multidisciplinary assessment

Evaluation of patients for TAVR starts with obtaining a thorough medical history and comprehensive review of systems and includes detailed physical examination along with imaging/ultrasound interrogation modalities of the cardiovascular systems such as transthoracic echocardiogram (TTE)/transesophageal echocardiography (TEE), cardiac catheterization study and a cardiac 64 multislice computerized tomography (CT) scanning. In certain cases, routine surface electrocardiography and hematologic and biochemical blood analyses may provide helpful information regarding the medical management of the patients during their perioperative period.

For patients who are determined to require a valve surgery but are at “high-risk” or inoperable, TAVR may be considered. Despite the favorable results of clinical trials and non-inferiority of TAVR to traditional AVR among patients with “intermediate risk”, TAVR is currently recommended only to patients who are identified as “high-risk” for surgery.^13^ Based on current consensus, only patients who satisfy the standard indications for AVR for AS but are deemed at “high-risk” for open-heart surgery undergo TAVR procedure. The indication for TAVR is only established after two Cardiac Surgeons have independently examined and found the patient as “too high risk to operate”. It needs to be noted that TAVR candidates should have a meaningful quality of life and a minimum life expectancy of at least one year.^14^ Typically, patients with Society of Thoracic Surgeons (STS) risk score of >10% or European system for cardiac operative risk evaluation score (EuroScore II, ver. 2011) of >20% are considered to be “high-risk”.^15^ This requirement may soon change in view of the results of new studies and ongoing clinical trials and TAVR may expand its indications to “intermediate-risk” patient population.

Once the patient is considered as appropriate candidate for TAVR; a multidisciplinary treating team discusses technical and anatomical feasibility of the procedure and set forth the strategy for TAVR. This care team consists of practitioners from interventional and non-interventional cardiology, cardiac anesthesiology, cardiac and/or vascular surgery and thoroughly evaluates the patient’s status and reassesses the risks and benefits for the proposed procedures. The choice between medical management and intervention (either in the form of TAVR or surgical AVR) is often a complex decision and frequently requires inputs from the multidisciplinary team.^16^ This team may acquire additional studies to measure the precise size and patency of the major vessels and perform multiple measurements of aortic valve with different modalities in order to choose the preferred vascular approach and suitable transcatheter valve type and size. Decisions to use alternative major vascular versus transapical approaches for deployment of the stent valves, are made by the multidisciplinary care team and generally rely on several pieces of information such as the presence of any plaque in left coronary ostium and the distance of coronary ostia from the aortic annulus.

## Anesthesiologist’s role in multidisciplinary care team

An anesthesiologist who is trained in cardiovascular medicine and has experience in caring for patients undergoing TAVR procedure is generally involved in the care of the patients as a member of the multidisciplinary team. The anesthesiologist discusses the available options and potential concerns regarding the anesthetic care with the rest of the treating team after a thorough independent assessment of the patients. Euroscore II and National Surgical Quality Improvement Program (NSQIP) risk calculator by the American College of the Surgeons are common risk assessment tools that are used by the anesthesiologists are while the STS scoring system is most commonly used by the cardiac surgeons for risk stratification. Risk assessment and constructing predictive models of prognosis and clinical outcome is an important part of preoperative assessment of the patients by the anesthesiologist.

## Medication reconciliation prior to TAVR

Perioperative beta-adrenergic blocking drugs should be continued in patients receiving this class of medications prior to the procedure. Continuation of the beta-adrenergic blockers on the day of TAVR or within 24 hours before the time of procedure is a Class I recommendation by the ACC/AHA guidelines. The dosage for beta-adrenergic blocking drugs should be titrated to heart rate and blood pressure prior to the procedure (Class IIa recommendation).

Statins should be continued in patients already taking statins (class I recommendation).^17^ Statins are also being considered as possible protective agents against perioperative mortality,^18^ acute kidney injury because of their anti-inflammatory and anti-oxidant effects as those with lower serum levels of high density lipoproteins are particularly prone to developing acute kidney injury after vascular surgery where significant amount of intravenous contrast material is used.^19^ Although there are no specific guidelines for pre-operative management of patients on anti-platelet therapy, most TAVR centers have different local policies to handle platelet inhibition during these procedures. These policies differ from one center to another. While in some institutes a full platelet inhibition is achieved by loading the patients with clopidogrel, some other keep anti-platelet therapy for postprocedural phase. It is the policy of our center to use antiplatelets only after the successful implantation of the valve.

Preoperative anemia is reported in more than half of the patients undergoing TAVR.^20^ Although preoperative anemia is an independent risk factor predicting an unfavorable outcome, periprocedural transfusion in an attempt to correct this anemia is also associated with significantly worse outcome than those who have not received blood during one-year follow-up.^20^ The association between perioperative transfusion and long-term survival as well as postoperative morbidities have also been shown in non TAVR procedures.^21^ Therefore, a restrictive use of blood transfusion is recommended though the criteria for blood transfusion yet needs to be determined.

## Intra-operative anesthetic management

The anesthetic technique most suited for the patient’s medical condition is decided by the anesthesiologist member of the care team and discussed with the rest of the members to entertain any potential concerns with any particular technique. The decision on the type of the anesthesia technique is generally based on preoperative co-morbidities and the procedural approach that will be used for TAVR. The patient’s preference is generally honored if it does not significantly interfere with the safety of the anesthetic care. If any conflict exists between the perceived risks and the patient’s wishes, it is the duty of the anesthesiologist to have a full conversation with the patients educating them on the risks and benefits of each particular technique. After providing a full discussion and explanation of the anesthetic procedures to the patients, a verbal agreement is generally reached between the anesthesiologist and the patients on the type of the anesthesia. In some institutes, a separate informed consent is signed by the patients for the anesthetic procedures that will be performed during TAVR, while some others may include the anesthesia consent into the global informed consent.

General anesthesia (GA) is the preferred choice of anesthesia by most anesthesiologists as well as the proceduralists. The advantages of include a definite control of airway and ventilation, minimal patient movement, easier management of hemodynamic challenges and more effective attenuation of stress response during the procedure, while providing more time for the proceduralists performing TAVR. It is also widely accepted by all patients especially those with claustrophobia, back pain or severe sleep apnea who have difficulty tolerating the supine position for a prolonged period of time. GA is also more favorable where TEE is needed. Indeed from the conception of TAVR, TEE has routinely been used along with GA in order to assess the valve deployment during the procedure. Additionally, a quick conversion is more feasible under GA when an emergent open surgical intervention becomes necessary to address serious periprocedural complications.^22^ The disadvantages of GA include dealing with difficult airway, challenges with ventilation in patients with chronic lung disease, absence of an awake patient’s responses used to monitor cerebral perfusion, myocardial depressant effects of anesthetics necessitating inotropic support ^23^ and delayed awakening from anesthesia.^24^

As the learning curve of the proceduralists reaches a new plateau and the techniques of TAVR evolve, the procedure time becomes shorter. Due to this improvement in the procedure time, more and more anesthesiologists feel comfortable in using monitored anesthesia care (MAC) with moderate sedation for patients undergoing TAVR. This anesthesia technique has emerged as a desired alternative anesthesia option depending on patients’ choice and comfort level. A unique beneficial aspect of MAC with moderate sedation includes the ability to continuously monitor the mental status for adequacy of cerebral perfusion.^25^ Behan et al. reported favorable outcome in a series of patients who underwent TAVR under remifentanil-based sedation.^25^ Subsequently, other observational studies compared the safety and feasibility of MAC for TAVR.^26,27^ In 2014, Fröhlich et al published a meta-analysis including studies comparing GA and MAC in patients undergoing transfemoral TAVR encompassing 7 observational studies with a total of 1542 patients altogether.^22^ In this study MAC was associated with a shorter hospital stay and a shorter procedure time. However, they were not able to show any statistically significant differences in cardiac and non-cardiac causes of 30-day mortality between MAC and GA.

Various agents and compounds including dexmedetomidine,^28^ remifentanil,^25^ midazolam and nalbuphine,^29^ ketamine and propofol^30^ have been used as monotherapy or in combination for sedating patients during TAVR.^31,32^ Table 1 shows the studies that examined pharmacologic characteristics of sedatives used in monitored anesthesia care for TAVR. Mayr et al in a review of 13 non-randomized studies and registries encompassing data from 3227 patients undergoing TAVR with sedation, report a rate of up to 17% for conversion to GA. The conversion to GA most commonly occurs due to vascular complications.^33^ In a recent multicenter prospective study, 310 pairs of patients undergoing TAVR with either MAC or GA were identified and matched for baseline characteristics. Though there was a concern regarding a possible increased risk of severe paravalvular regurgitation and need for permanent pacemaker in MAC group, similar immediate and late outcome was reported.^34^ This might be due to the more liberal use of TEE in GA group. Prospective randomized clinical trials are anticipated to clarify the questions surrounding the superiority of either technique or any specific sedative selected for MAC.

**Table 1:** Studies that examined pharmacologic characteristics of sedatives used in monitored anesthesia care for TAVR

**Author (year)**	**Design**	**Agent(s)**	**No. of patients**	**Summary of findings**
Behan (2008)	MAC vs. GA	remifentanil	9/3	No significant differences in procedural success, procedure time, or hospital stay between the two groups
Dehdin (2011)	MAC vs. GA	ketamine+ propofol	34/91	Less intraoperative hemodynamic instability and significant shortening of the procedure and hospital stay in MAC
Durand (2012)	MAC	midazolam+ nalbuphine	151	Conversion to general anesthesia was required in 3.3% and was related to complications. The combined-safety endpoint was reached in 15.9%
Ben-Dor (2012)	MAC vs. GA	ketamine + propofol or dexmedetomidine	22/70	MAC associated with shorter procedure time and in-hospital length of stay
Motloch (2012)	MAC vs. GA	midazolam+ remifentanil	33/41	MAC was as safe as GA, total procedure time was shorter and patients could be mobilized significantly earlier in MAC group
Yamamoto (2013)	MAC	propofol + remifentanil	44/130	Intensive care unit stay and hospital stay were longer in GA group, Conversion to general anesthesia was required in 4.6%
Park (2014)	MAC (cases)	dexmedetomidine	2	MAC with dexmedetomidine was feasible
D’errigo (2016)	MAC vs. GA	various agents	310/310	similar immediate and late outcome

The ideal setting for a TAVR procedure suite is a hybrid operating room, fully equipped with fluoroscopy bed and devices, and a standby team including clinical perfusionist and cardiac surgeon in case of need for an emergency cardio-pulmonary bypass and conversion to open-heart surgery.^35^ Invasive monitoring as well as large bore peripheral and central access is required through the procedure. However the insertion of pulmonary artery catheter is usually at the discretion of the anesthesiologist and is preferred in the presence of pulmonary hypertension or left ventricular dysfunction. Transcutaneous defibrillation pads should be in place. Additionally, transvenous pacing wires need to be inserted and tested. In case of need for TEE during the case GA may be necessary.

Ultrasound imaging technology with appropriate probes for TEE and epicardial/intra-cardiac echocardiography, high-resolution fluoroscopy and contrast angiography have been used alone or together as a guide during the procedure. In addition to providing important information regarding regional wall motion abnormalities and left ventricular function, TEE is invaluable in cases with inadequate angiographic resolution. However, angiographically guided procedures have been shown to have similar clinical outcomes compared to those performed under TEE guidance.^36^ Ferrari et al. demonstrated that, in transapical approach, TEE serves well as the primary guidance tool.^37^ Though TEE probe may interfere with fluoroscopy and withdrawn at times.

Postprocedural TEE study should include measurements of mean and peak gradient as well as effective orifice area, assessing transvalvular and paravalvular leak and ensuring that the prosthetic valve is well seated to prevent dislodgement or significant aortic insufficiency.^38^ The European Association of Echocardiography along with the American Society of Echocardiography has published a recommendation for the use of echocardiography in new transcatheter interventions for valvular heart disease.^39^ Undersized stent valves, malpositioning of the valve, excessive asymmetric calcification of the native valve or congenital abnormalities could lead to post TAVR paravalvular leaks.^40-42^ However, the occurrence of paravalvular leak has decreased due to the availability of different valve sizes, increased operator experience and improved preoperative measurements.^43^

The goals of maintenance of GA during TAVR would include maintaining the preload, heart rate control in low normal ranges in order to increase diastolic filling time, and treatment of hypotension with direct alpha agonist vasopressors to prevent tachycardia.^44^ Rapid ventricular pacing (RVP) is used for cardiac standstill and decreasing left ventricular ejection against inflated balloon for Sapien Valves.^45^ Hemodynamic support during and after RVP is essential^46^ and it is recommended to maintain mean arterial pressure above 75 mmHg prior to the initiation of RVP to avoid prolonged hypotension.^47^ In our current practice we also increase the depth of sedation during RVP to maximize patient comfort. Though spontaneous return of sinus rhythm upon cessation of RVP is common, sustained ventricular tachycardia or ventricular fibrillation necessitating defibrillation could happen occasionally specially in the setting of preexisting left ventricular dysfunction.^48^ Prior to the deployment of the valve, anti-coagulation is achieved by heparin and is controlled with activated clotting time. For the case of Sapien valves, once the valve has been deployed, retraction or repositioning after balloon expansion is not possible. For the cases of malpositioned valves, the only option would be deployment of another percutaneous valve in through the first malpositioned valve, the so-called ‘valve-in-valve’ technique. In contrast CoreValve can be gradually deployed and at any point of deployment it could be retrieved and re-deployed. Final fluoroscopic and angiographic imaging is performed before the valve stent delivery catheter is removed. Arterial puncture is closed by endovascular or open surgical vascular suture. Any sudden change in hemodynamic status should alert the team about the possibility of vascular injury. Retroperitoneal hemorrhage is an uncommon but grave complication of transfemoral percutaneous approach, which could have few external manifestations even in the setting of large volume blood loss. Aggressive volume replacement and vasopressor support are needed to maintain coronary perfusion until the damage is repaired and bleeding is controlled. Placement of endovascular occlusion balloon from contralateral side, iliac stenting and open surgical repair are among the options in the case of vascular perforation.

Depending on the type of the valve, vascular approach and proceduralist’s experience a few complications could arise. Coronary artery occlusion could occur due to the folding of native calcified valve or malpositioning of the transfemoral stent valve. Continuous and vigilant monitoring of the patient during the procedure and immediate availability of cardio-pulmonary bypass team, intra-aortic balloon pump and left main coronary stenting system is necessary. CoreValve prostheses are more commonly associated with the occurrence of post-procedure atrioventricular block compared to SAPIEN valves.^49^ Epicardial electrodes are ultimate pacing options in transapical approach for stent valve replacement. Cardiac tamponade is also another complication that could happen during cannulation of aorta, wire and prosthetic stent valve insertion through the major vasculature, perforation of myocardial wall during insertion of pacing wires and development of left ventricular apical pseudo-aneurysm and its progression to tamponade in transapical approach.^50^

## Postoperative anesthetic management

Patients are transferred post-operatively to a closely monitored area for continuation of the care; however, fast tracking from recovery room to a step-down unit with telemetry monitoring has been exercised in some centers. Pain control is generally achieved with various agents including non-steroidal anti-inflammatory drug, acetaminophen and low dose narcotics. An immediate post-operative TTE will be obtained recommended if TEE is not used during the procedure.

Bagur et al looked into the incidence and predicting factors of acute kidney injury (AKI) following TAVR, and compared AKI’s occurrence in TAVR vs. surgical AVR. They reported that among patients with chronic kidney disease, who underwent TAVR, AKI rate was significantly lower than surgical AVR.^51^ Crowhurst et al examine the frequency of AKI in 209 undergoing TAVR. A total of 82 patients have demonstrated ≥0.3 mg increase in serum creatinine postoperatively. These also identified hypertension, blood transfusion and chronic obstructive pulmonary disease as predicting risk factors for AKI after TAVR.^52^ Van Linden et al report a linear increase in the incidence of AKI with the volume of contrast material used during transapical TAVR.^53^ Additionally, in view of the potential risk of renal injury, ensuring adequate preoperative hydration, limiting the amount of injected contrast agent, using hypo-osmolar contrast have been advocated to limit kidney injury. RenalGuard system, a novel strategy for maintaining euvolemia and inducing a vigorous diuresis, was shown to reduce the risk of acute kidney injury in patients undergoing TAVI.^54^

The prevalence of neurologic impairments after TAVR has been investigated and the rate of clinically silent cerebral emboli were reported to be high. This phenomenon is also observed among patients undergoing transapical TAVR approach. Despite prevalent detection of embolic events by MRI; patients were clinically asymptomatic, or affected with a quick resolution of symptoms.

## Summary

TAVR is rapidly gaining popularity as new devices in diverse size, shape and flexibility are developing. TAVR soon may become the standard of care for a subdivision of patients with severe AS expanding its indications from “high-risk” group of patients to those with “intermediate-risk”. As an anesthesiologist; understanding the procedure and the challenges inherent to it is of paramount importance, in order to implement optimal care for this “high-risk” population undergoing a novel procedure.

## Ethical Approval

Not applicable.

## Competing of interests

None to be declared.
